# Overexpression of N-Myc Downstream-Regulated Gene 2 (NDRG2) Regulates the Proliferation and Invasion of Bladder Cancer Cells *In Vitro* and *In Vivo*

**DOI:** 10.1371/journal.pone.0076689

**Published:** 2013-10-16

**Authors:** Ruixiao Li, Chuigong Yu, Feng Jiang, Lei Gao, Jianying Li, Yingmei Wang, Noor Beckwith, Libo Yao, Jing Zhang, Guojun Wu

**Affiliations:** 1 Department of Biochemistry and Molecular Biology, State Key Laboratory of Cancer Biology, the Fourth Military Medical University, Xi’an, China; 2 Department of Urology, Xijing Hospital, the Fourth Military Medical University, Xi’an, China; 3 Department of Gynaecology and Obstetrics, Tangdu Hospital, the Fourth Military Medical University, Xi’an, China; 4 Department of Pathology, the Fourth Military Medical University, Xi’an, China; 5 Harvard Medical School, Boston, Massachusetts, United States of America; University of Torino, Italy

## Abstract

N-Myc downstream-regulated gene 2 (*NDRG2*) is a candidate tumor suppressor gene, which plays an important role in controlling tumor growth. The aim of this study was to investigate the expression of *NDRG2* gene in bladder cancer (BC) tissues and several bladder cancer cell lines, and to seek its clinical and pathological significance. Ninety-seven bladder carcinoma and 15 normal bladder tissue sections were analyzed retrospectively with immunohistochemistry. The human bladder cancer cell line T24 was infected with LEN-*NDRG2* or LEN-LacZ. The effects of *NDRG2* overexpression on T24 cells and T24 nude mouse xenografts were measured via cell growth curves, tumor growth curves, flow cytometric analysis, western blot and Transwell assay. *NDRG2* was highly expressed in normal bladder tissue, but absent or rarely expressed in cacinomatous tissues (χ^2^=8.761, p < 0.01). The *NDRG2* level was negatively correlated with tumor grade and pathologic stage(r=-0.248, p < 0.05), as well as increased c-myc level (r=-0.454, p< 0.001). The expression of *NDRG2* was low in the three BC cell lines. T24 cells infected with LEN-*NDRG2* showed inhibition of proliferation both *in vitro* and *in vivo*, and *NDRG2* overexpression can inhibit tumor growth and invasion *in vitro*.

## Introduction

The incidence of bladder cancer is increasing. An estimated 386,300 new cases and 150,200 deaths from bladder cancer occurred in 2008 worldwide [1]. In men, bladder cancer is the fourth most common cancer only after prostate, lung, and colorectal cancers, accounting for 7% of all cancer cases in USA [2]. In more than 75% of the cases, the diagnosis is made at an early stage of disease (stages Ta and T1). Despite having received adequate treatment, the five-year overall survival rate for pathologic T2 disease is 52–77%, T3 disease 40–64%, and T4 or lymph node-positive disease 26–44% [3]. There are numeral therapeutic interventions to treat this disease; however, the overall survival rate has not been improved in the last twenty years. Therefore, to establish a new mode of therapy using antioncogene agent to improve the survival rate of patients is highly desirable.

The N-myc downstream regulate gene 2 (*NDRG2*) belongs to the NDRG family, which includes four members: *NDRG1, NDRG2, NDRG3* and *NDRG4*. The sequence homology within the human *NDRG* family is 57-65% and the *NDRG* family has been investigated in some of human cancer and nervous system disorders [4]. As a gene to regulate downstream of Myc, *NDRG2* expression has been confirmed to be reduced in many types of carcinomas, including thyroid cancer, liver cancer, meningioma, pancreatic cancer and prostate cancer [5–11]. These studies suggest that *NDRG2* might play an important role in controlling morbidity of carcinomas. The *NDRG2* has been confirmed to be involved in cell growth and differentiation, meanwhile, *NDRG2* expression in high-grade gliomas has been shown to associate with survival [12,13].

**Figure 1:**
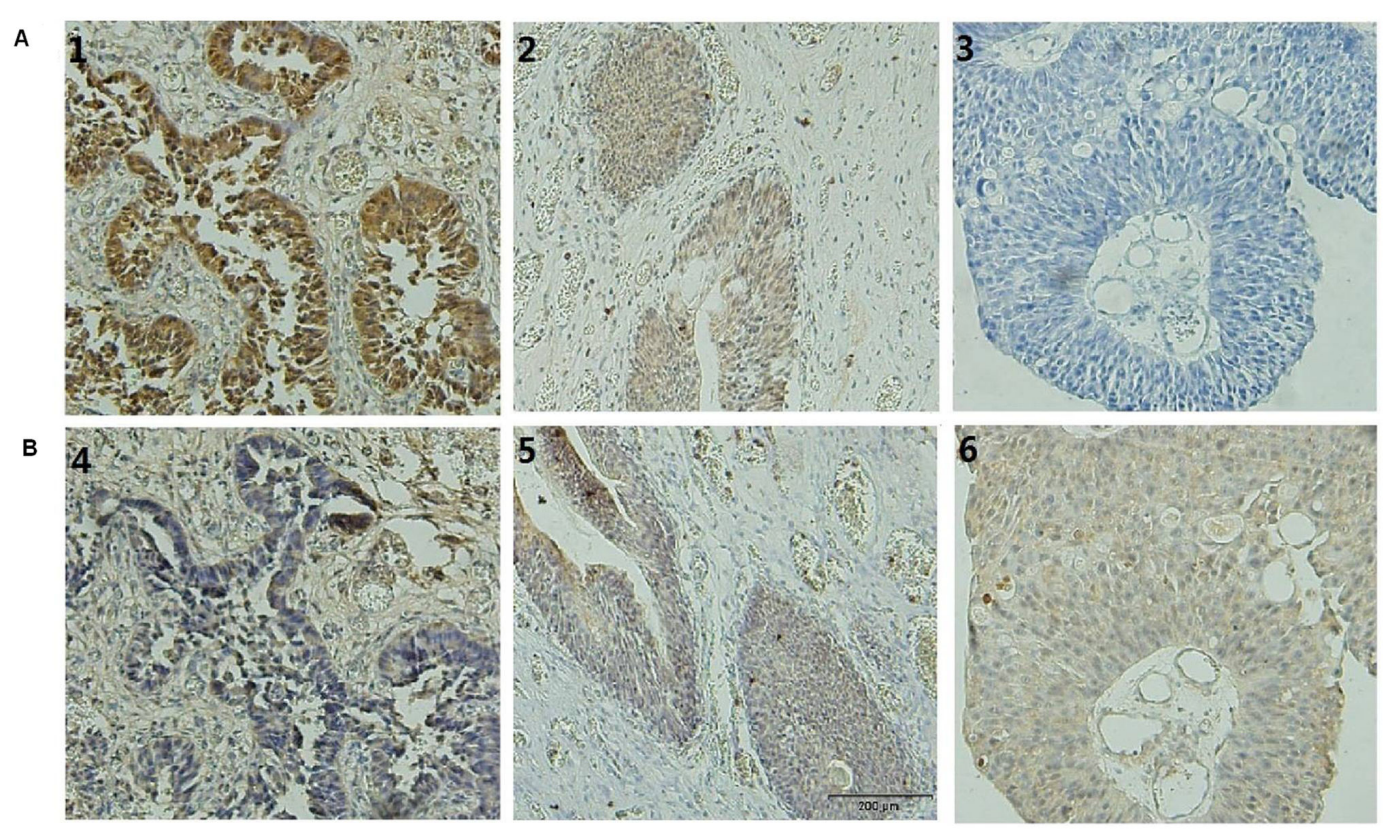
The expression of *NDRG2* and c-Myc protein in bladder carcinoma tissues detected with immunohistochemistry staining (A) The expression levels of *NDRG2* protein decrease while the degree of malignancy of bladder carcinoma increases (x400)(1). Normal bladder tissue; (2) Bladder papilloma(T0-Ta); (3) High-level bladder cancer(T2-T4). (B) The expression levels of c-Myc protein increases while the degree of malignancy of bladder carcinoma increases (x400)(4). Normal bladder tissue; (5) Bladder papilloma(T0-Ta); (6) High-level bladder cancer(T2-T4). The sections of (B) (4–6) originated from the same paraffin blocks as (A) (1–3) respectively.

Though several studies have investigated the function of *NDRG2* in common tumors, there has not been functional characterization of the potential role of *NDRG2* gene in bladder cancer. Therefore, the aim of the current study was to investigate the role of *NDRG2* in human bladder cancer. First, we examined the expression of *NDRG2* in human bladder carcinoma tissues and compared to normal bladder tissues by immunochemistry. We found that the expression level of *NDRG2* in BC tissues was lower than that in normal tissues. Next, we used bladder cancer cell lines as models to evaluate the effect of *NDRG2* on tumor growth, differentiation and invasion *in vitro* and *in vivo*. Our results suggest that *NDRG2* has a potential antioncogenic role in bladder cancer.

## Materials and Methods

### Clinical Samples

Formalin-fixed and paraffin-embedded blocks of bladder carcinoma tissues were randomly collected from 112 patients and controls (mean age 63.4 years, range from 21 to 81 years) from the Department of Urologic Surgery, Xijing Hospital, FMMU (Xi’an, China) between 2008 and 2011.These samples comprised 15 normal bladder tissues, 20 bladder papilloma (T0-Ta), 38 low-level bladder cancer(Tis-T1) and 39 high-level bladder cancer samples(T2-T4). Written consent was obtained from each subject. This study was approved by the Ethics Committee of Xijing Hospital, Xi’an, China.

**Figure 2:**
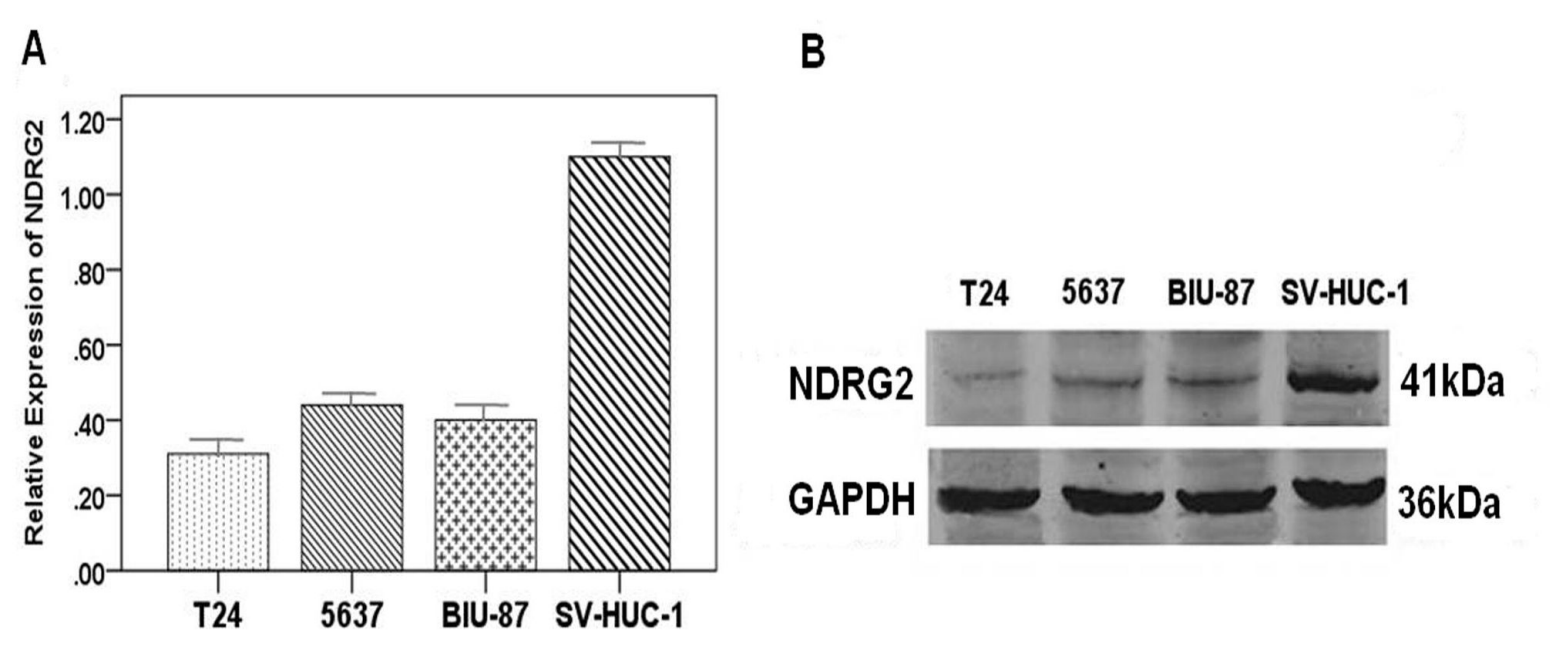
Differential expression of *NDRG2* among bladder cancer cell lines (A) Real-time PCR analysis of *NDRG2* mRNA expression. (B) Expression level of *NDRG2* protein as assayed by western blot. All the assays were repeated independently for at least three times. The results are shown as the mean ± SD (** p<0.001)..

### Immunohistochemistry (S-P: streptavidin-perosidase)

Mouse anti-human *NDRG2* monoclonal antibody and c-Myc monoclonal antibody were purchased from Santa Cruz Biotechnology Company (Santa Cruz, USA.). The immunohistochemistry kit was purchased from the Boster Company (Wuhan, China). The immunohistochemistry staining was performed according to the manufacturer’s instruction. The following preparations were made from each tissue block: a slide stained with HE (the pathologic stage were checked by pathologists), a slide incubated with anti-*NDRG2* antibody, a slide incubated with c-myc antibody.

In order to accurately determine the positive expression and to minimize the false-positive rate, we used two arbitory semi-quantitative scoring system to evaluate the extent and intensity of the staining by three pathologists independently. The scores were defined as follows: (1) the extent of staining scoring: 0 point for staining <5%, 1 point for staining 6% to 25%, 2 point for staining 26% to 50%, 3 point for staining 51% to 75%, and 4 point for staining > 75%(2). The intensity of staining scoring: 0 point for negative staining, 1 point for weakly positive staining, 2 points for moderate staining and 3 points for strong staining (tan). For each specimen, the scores derived from the two scoring systems were multiplied. The results of determination were divided into four levels: negative (0 to 1, -), weakly positive (2 to 4, +), positive (5 to 8, + +), strong positive (9 to 12, + + +). Imaging was performed by light microscopy (Olympus, Nagano, Japan) and calculated with statistical analysis.

### Cell Culture

We selected human T24, 5637 and BIU-87 cells lines to be used in the current investigation. These cell lines have been previously confirmed as the appropriate cell lines for the research of bladder cancer. As normal control, we chose human bladder cell (SV-HUC-1). All the cell lines were purchased from the Cell Bank, Chinese Academy of Sciences, Shanghai, China. The cells were cultured in RPMI1640 (Gibco) supplemented with 10% fetal bovine serum (Sijiqing Hangzhou, China). All the cell lines were cultured in sterile conditions at 37°C and 5% CO_2_.

**Figure 3:**
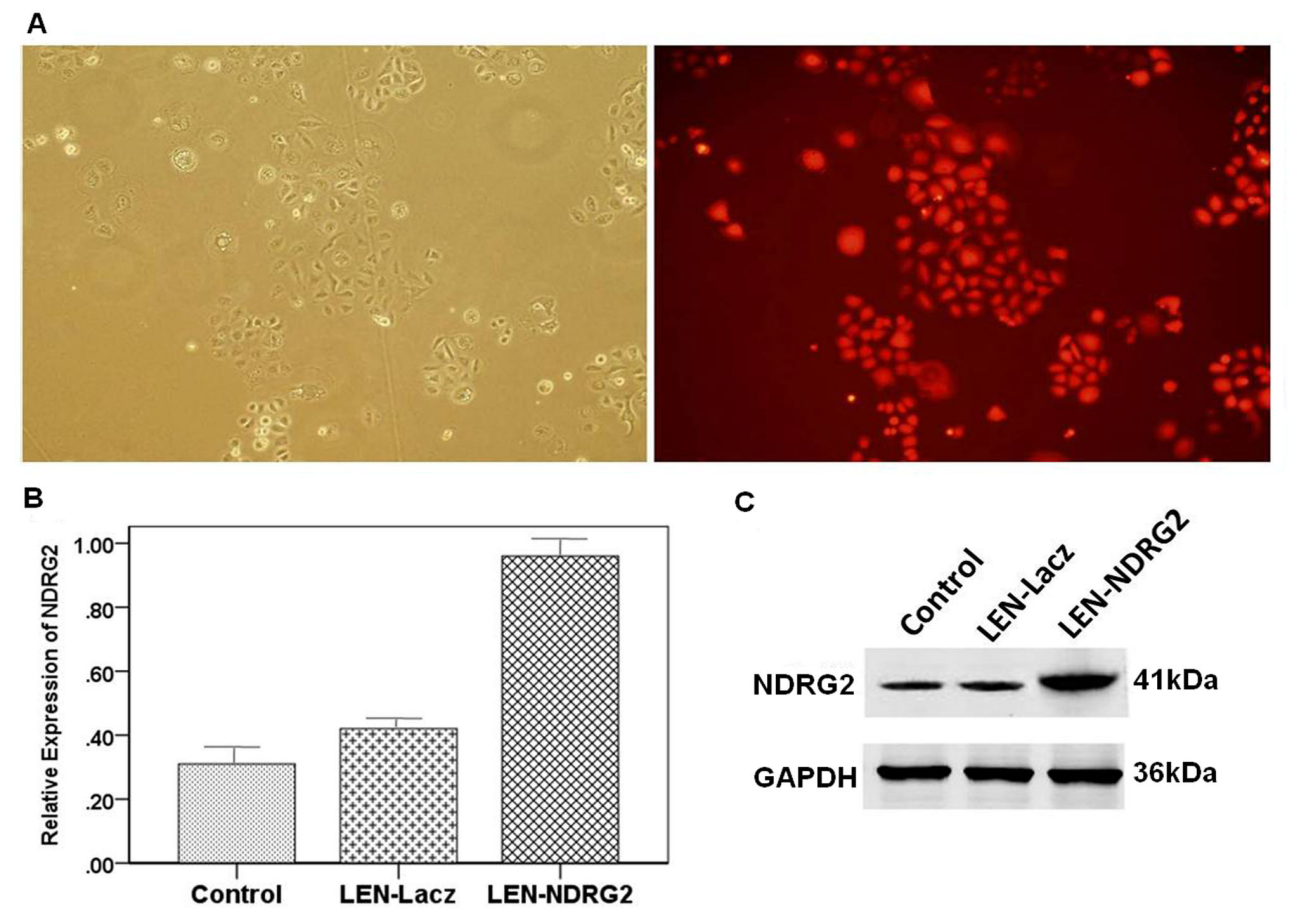
pLEN-GFP-*NDRG2* infected T24 cells to increased *NDRG2* expression in bladder cancer cells (A) Detection of lentiviral infection efficiency, and phase contrast (left) or GFP (right) images were obtained four days after infection. (B) Real-time PCR analysis of *NDRG2* mRNA expression in T24 cells. (C) Western blot analysis of *NDRG2* protein expression. The results are shown as the mean ± SD (** p<0.01)..

### Real-time Quantitative PCR

The total RNA was extracted from cells using TRIzol reagent (Takara Bio, Japan) and reverse transcribed using M-MLV Reverse Transcriptase (Fermentas) according to the manufacturer’s instruction. The primers used were as follows: GAPDH, 5′-AGGTCCACCACTGACACGTT-3′ and 5′-GCCTCAAGATCATCAGCAAT-3′; *NDRG2*, 5′-GCCCAGCGATCCTTACCTACC-3′ and 5′-GGCTGCCCAATCCATCCAACC-3′. The amplification program consisted of polymerase activation at 95°C for 30 seconds and 40 cycles of denaturation at 95°C for 15 seconds, annealing and extension at 59°C for 30 seconds. The relative expression levels were normalized using the comparative threshold cycle (2 ^-∆∆Ct^). The Real-time PCR performed by using CFX96 Touch PCR system (Bio-Rad). All above mentioned experiments were repeated at least three times.

### Western Blotting Analysis

All the cells were collected in exponential phase and then the total protein was extracted with lysis buffer containing 1% Tween 20, and finally quantitated with BCA assay. Equal amount of proteins 20µg were subjected to 10% concentration SDS-PAGE. The proteins separated by SDS-PAGE within the gels were transferred onto nitrocellulose (NC) membranes (Amersham, St. Giles, UK). Subsequently, the NC membranes were blocked with 5% non-fat milk for 1h at room temperature and then incubated with mouse anti-human *NDRG2* antibody (1:800) (Santa Cruz, USA) overnight at 4°C. GAPDH protein detection was used as an internal control. After washing three times, the transferred blots containing *NDRG2* bands were visualized with horseradish-peroxidase (HRP)-conjugated anti-mouse or anti-rabbit secondary antibody (dilution 1: 2000, Santa Cruz) for 2 h at room temperature. The enhanced chemiluminescence (ECL) system detection solutions (Pierce, NJ) were then applied, and quantified by Kodak Digital Science ID software (Kodak, NY).

### Lentivirus Infection

To produce lentivirus, 293T cells were co-transfected with pLEN-GFP-*NDRG2* or pLEN-GFP-Lacz, which were amplified in *E. coli* DH5, purified using a Plasmid Maxi Kit (Qiagen, Valencia, CA), and transfected into 70% confluent 293T cells using lipofectamine 2000 (Invitrogen). Lentiviral particles were harvested from the supernatant 72 hours after transfection and purified by ultracentrifugation. These particles were hereafter referred to as LEN-*NDRG2*, and LEN-Lacz (negative control). Stably infected T24 cells were selected using blasticidin, by counting green fluorescent protein (GFP)-positive cells under fluorescence microscopy (Olympus, Japan), applied at the minimum concentration of blasticidin, after a serial titration, required to kill uninfected T24 cells. The cell line was divided into the following three experimental groups: 1) *NDRG2* group (LEN-*NDRG2*-infected cells), 2) Lacz group (LEN-Lacz-infected cells), and 3) CON group (non-infected cells). Both Real-time PCR and Western blot confirmed that we successfully infected T24 cells with the lentiviral and overexpression of *NDRG2* properly after infection. We also ascertained the differential expression of proteins that correlated with cell cycle stage, apoptosis and cell migration and invasion (cell cycle protein: cyclinD1, CDK4; cell apoptosis protein: p21; cell migration and invasion protein: MMP-2 and MMP-9).

### Cell Growth Inhibition Tests *in Vitro*

This test included MTT assays, Colony formation assays and flow cytometric analysis (FCA) (BD Biosciences, NJ). Each assay was applied to each of the three groups (the blank control; LEN-LacZ, the negative control; and LEN-*NDRG2*, the experimental group). The experiments were repeated five times(1). MTT assay: All the cells, including those infected, were grown in exponential phase and detached by trypsin treatment. Viable cells (2000 cells/ml) were inoculated into 96-well plates and every group had six reduplicative wells. At different time points, MTT reagent was added 20 μl/well (5mg/ml) and incubated at 37 °C for 4 h. The reaction was stopped by the addition of 150 µl dimethyl sulfoxide (DMSO) followed by shaking for 10 min. Absorbance (A) values were measured with an autokinetic enzyme scaling meter (Bio-Rad, USA) at 490 nm wavelength. Cell growth curves then were then drawn based on the average A values(2). Colony formation assay: Cells were seeded into six -well plates (200 cells /well) (in three duplicate wells) and cultured at 37°C in 5% CO_2_. After two weeks, the cells were fixed with methanol for 20 min and then stained with Giemsa for 20 min. ddH_2_O was used to wash the cells three times to obtain a clean background. The number of colonies and the cell number in each colony were counted and statistically analyzed(3). FCA: T24 cells (1 x 10^6^ cells/ well) were plated in six-well culture dishes with lentivirus infected LEN-*NDRG2*. Three groups of cells were collected after 48h and 72h respectively and finally centrifuged and fixed in 95% ethanol. After overnight incubation at 4°C, cells were stained with 0.5% propidiumiodide (10µl) in the presence of 0.01% RNAse A and 0.1% Triton X-100, then were measured with a flow cytometer.

**Figure 4:**
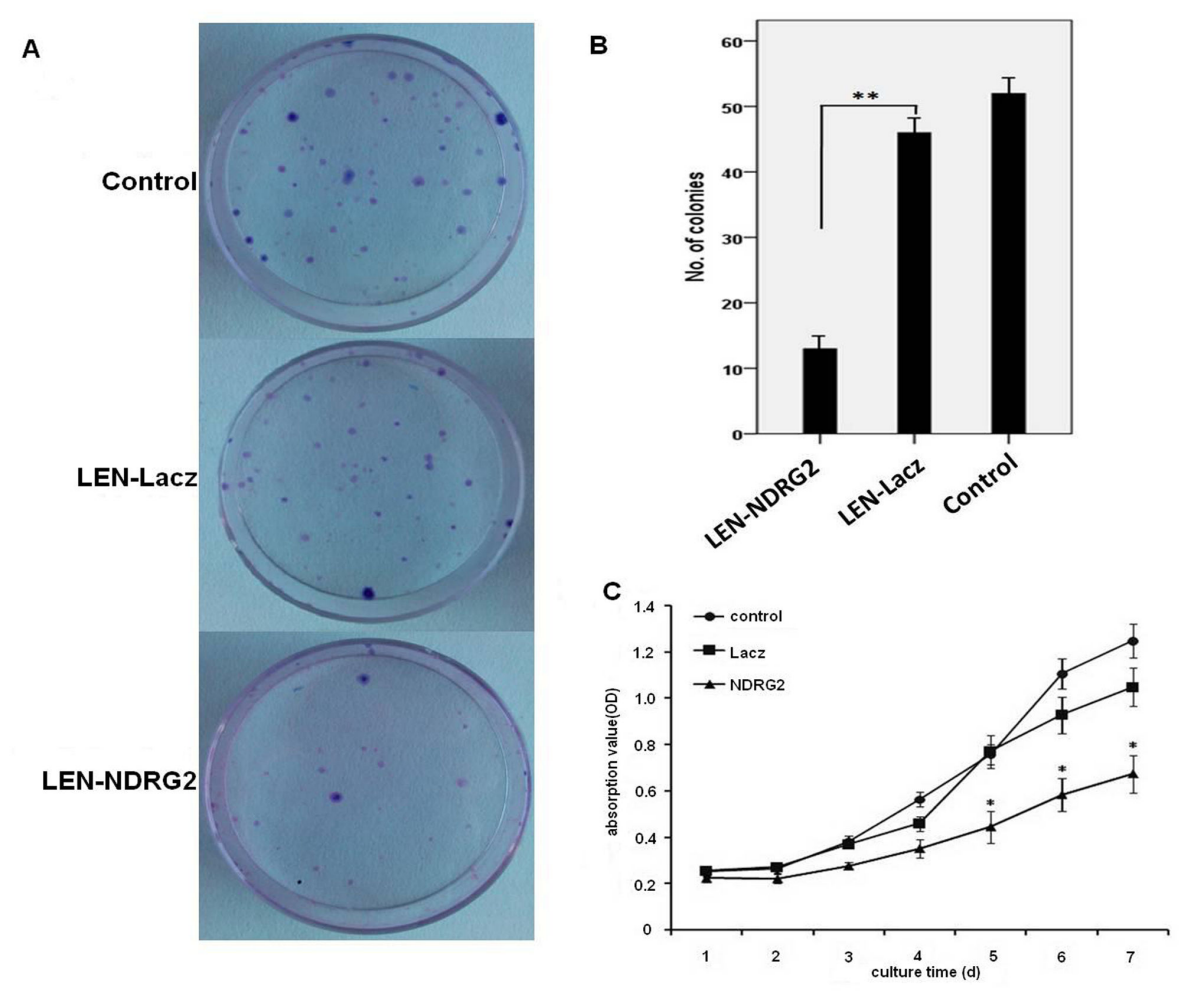
*NDRG2* overexpression inhibits proliferation of human bladder cancer cells. (A and B) Colony formation assay. Upregulation of *NDRG2* inhibits colony formation. (C) The cell growth curves of T24 cells by MTT method. All the assays were repeated independently for at least three times. The results are shown as the mean ± SD (*=p<0.001).

### Migration and Invasion Assays

Invasion assay with a Matrigel-coated membrane and migration assay with no Matrigel membrane were performed using a 24-well Transwell inserts (8µm pore filters, BD Biosciences, Bedford, MA), which were performed according to manufacturer’s instructions. The exponential phase cells were harvested and resuspended (2×10^5^ cells/ml) in serum-free medium, then seeded and resuspended in 200µl culture medium in the upper chamber. Next, culture medium with 20% FBS was placed in the bottom well. The Transwell were incubated at 37°C and 5% CO_2_ atmosphere over night. The T24 cells were allowed to migrate through a porous to the bottom of the membrane. After incubation, the cells on the bottom of the membrane were fixed with methanol for 5 min and then stained with Kristallviolet. The number of invading cells or migrating cells was determined by microscope at a 400x magnification on each membrane and calculated the mean number of cells per field. All the assays were repeated at least three times independently.

### Growth Inhibition Assays *in Vivo*

Six-week-old male BALB/c nude mice were purchased from Shanghai Experimental Animal Center, Shanghai, China. The LEN-*NDRG2* group and LEN-Lacz group cells were harvested and resuspended with 1 × PBS and the concentration was adjusted to 5 × 10^7^ per ml, 200µl cell solution (1 x 10^7^ cells) were injected subcutaneously into the left flanks of the nude mice. The mice were randomly divided into two groups: LEN-*NDRG2* and LEN-LacZ (n=5 per group). When the mean size of inoculated tumors reached to 300 mm^3^ (as calculated by the equation: V [mm^3^] = ab^2^ /2) *in vivo*, the mice were sacrificed by cervical dislocation and tumor specimens were collected, photographed and measured for their volumes and weight. The mice were anatomized to check whether there were metastasis to other organ and lymph node. The expression of *NDRG2* protein in the inoculated tumors of nude mice was detected by western blot. Other markers for protein expression were also measured, including cell cycle protein: cyclinD1, CDK4; cell apoptosis protein: p21; cell migration and invasion protein: MMP-2, MMP-9.

**Figure 5:**
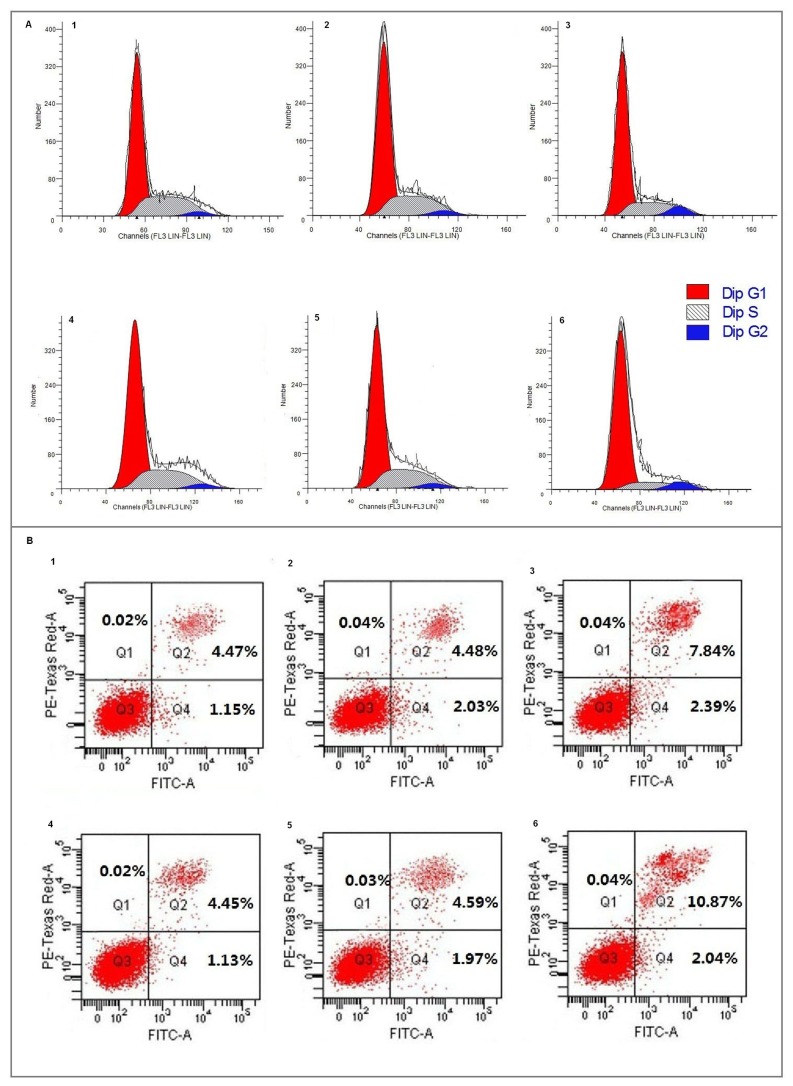
FCA of cell cycle arrest and apoptosis induced by overexpression of *NDRG2*. (A) T24cells infected with LEN-*NDRG2* lentivirus were more obviously arrested in G0/G1cycle. (B) Percentages of apoptotic cells in LEN-*NDRG2* groups were greater than in the other two groups(1–3). 48 h after the infection (4–6); 72 h after the infection; (1 and 4) blank control (PBS); (2 and 5) LEN-LacZ; (3 and 6) LEN-*NDRG2*. All the assays were repeated independently for at least three times. The results are shown as the mean ± SD.

**Table 1:** The Expression of NDRG2 in normal bladder tissue and bladder carcinoma tissues.

Group	NDRG2(-)	NDRG2(+)	*X* *^2^* *value*	*p value*
**Normal tissue**	3	12		
**Bladder carcinoma**	59	38	8.761	**<0.01**

Data are presented as n (number of samples). Statistical significance was evaluated with χ^2^test.

### Statistical Analysis

Statistical analyses were performed using SPSS17.0 software (SPSS company, IN). All data are represented as the mean ± standard error derived from at least three independent experiments. For immunohistochemistry, differences across groups were validated using Chi-square tests and Pearson correlation. Analysis of variance (ANOVA), Student-Newman-Keuls (SNK) tests and non-parametric test were performed to determine whether there were differences among the results of assays *in vitro* and *in vivo* assays. A *P*-value less than 0.05 were considered statistically significant.

## Results

The frequency of positive expression of *NDRG2* in bladder carcinoma tissues was 39.17% (38/97), which was lower than that in normal bladder tissues (80%, 12/15), (χ^2^= 8.761, *P*<0.01) (Table 1). *NDRG2* expression, both frequency and levels, was not correlated with gender or age, but was inversely correlated with pathologic stage (r=-0.248, *P*<0.05) (Table 2). Immunohistochemistry showed that *NDRG2* protein positive was staining in the cytoplasm of normal tissues and bladder carcinoma (Figure 1A). The expression levels of *NDRG2* in bladder carcinoma were inversely correlated with c-Myc in bladder carcinoma (r=-0.454, *P*<0.001) (Figure 1B).

**Figure 6:**
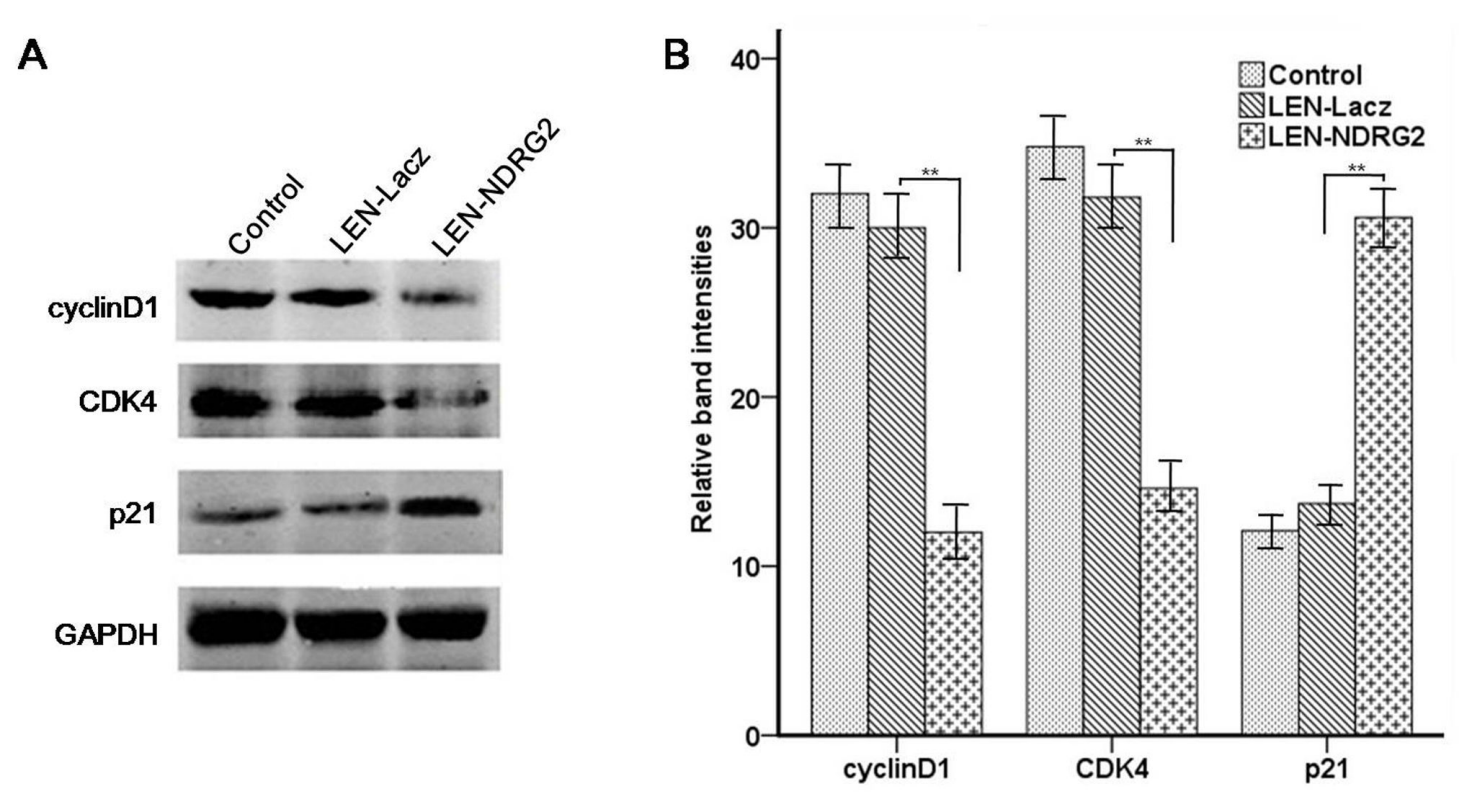
The influences of *NDRG2* overexpression on T24 cells infected with lentivirus. (A) The effect of *NDRG2* overexpression on G1 cell cycle and apoptosis regulators as assayed by western blot. (B) Relative quantification of protein expression, normalized to GAPDH levels. The figure shows the tendency of each group as indicated. All the assays were repeated independently for at least three times. The results are shown as the mean ± SD (** p<0.01)..

**Table 2:** The Relationship between the expression of NDRG2 and bladder carcinoma characteristics.

**Clinicopathological**		**Expression level of NDRG2**				***r-value***	***p-value***
Features		−	+	++	+++		
Gender							
	Male	40	11	7	3		
	Female	19	12	5	0	0.32	0.759
Age							
	≤60years	23	10	6	1		
	>60years	36	13	6	2	-0.049	0.637
Grade							
	Papilloma	10	4	4	2		
	Low	22	9	6	1		
	High	27	10	2	0	-0.248	0.014

Data are presented as n (number of samples). Statistical significance was evaluated with Pearson correlation coefficient test.

Both western blot and Real-time PCR showed that all three BC cell lines had lower expression levels of *NDRG2* protein and mRNA than the normal human bladder cells (SV-HUC-1). Among the three BC cell lines, the T24 cells showed the lowest expression levels(Figure 2A, B).

**Figure 7:**
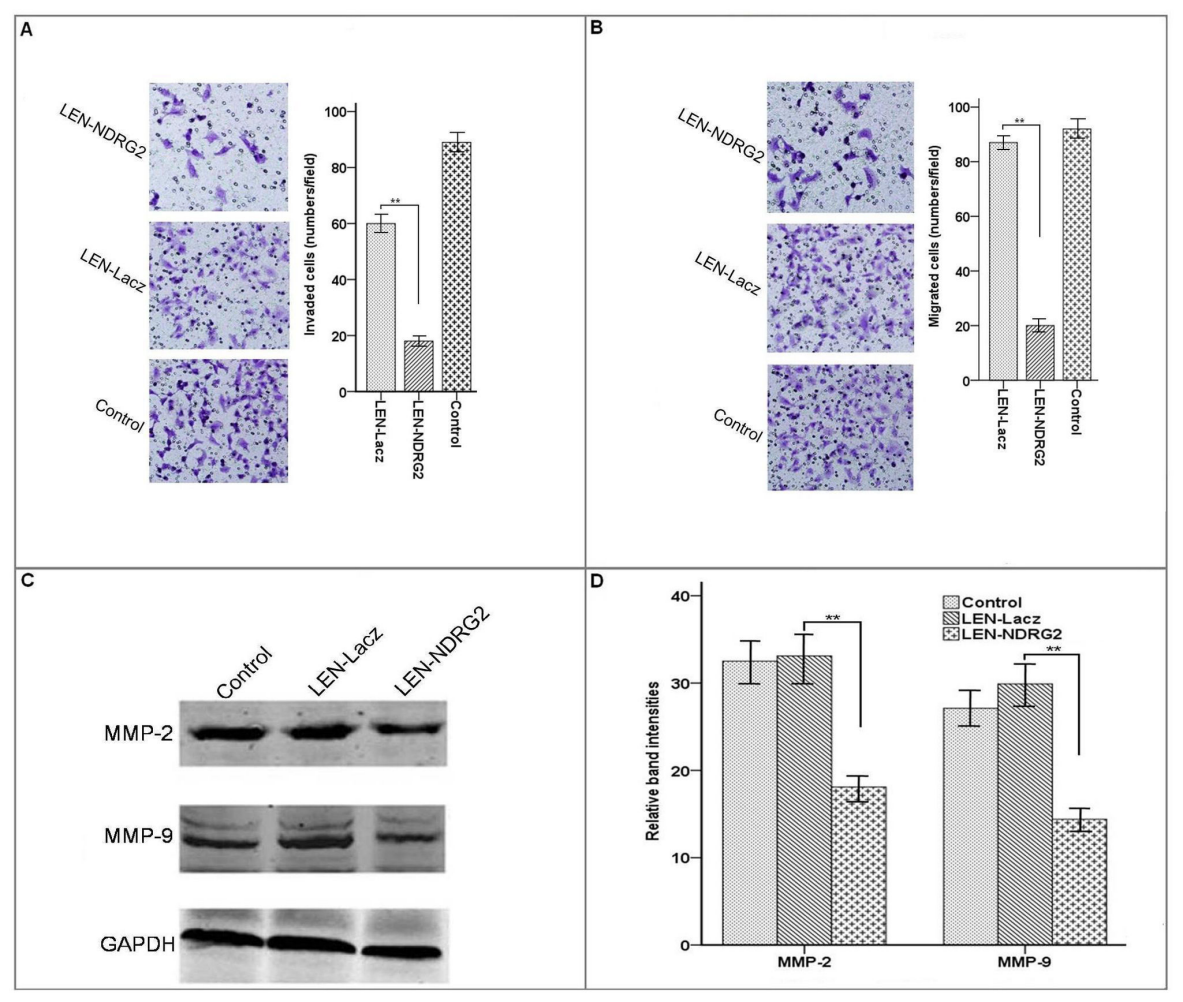
Overexpression of *NDRG2* suppresses human bladder cancer cell invasion, migration and expression of MMP-2 and MMP-9. (A) Invasion analysis of T24 cells treated with LEN-*NDRG2*. The invasion ability was estimated using Transwell coated with Matrigel. The invasiveness of LEN-*NDRG2* group cells was significantly lower than that in the other two groups. **p<0.001. (B) Migration analysis of T24 cells treated with LEN-*NDRG2*. The migration ability was estimated using Transwell uncoated with Matrigel. The migration ability of LEN-*NDRG2* group cells was significantly lower than that in the other two groups. **p<0.001. (C and D) Expression of MMP-2 and MMP-9 were measured after T24 cells were infected with LEN-*NDRG2*. LEN-*NDRG2* group reduced MMP-2 and MMP-9 expression compared to the other groups (** p<0.01).

**Table 3:** The proportion of Cell cycle phase and cell apoptosis induced by LEN-NDRG2 on T24 cells.

Group		G0/G1	G2/M	S	Apoptosis
**Control**	**48h**	62.42±2.05	3.55±0.55	34.03±1.65	5.53±0.62
	**72h**	69.84±2.60	2.79±0.44	27.73±0.90	5.57±0.59
**LEN-LacZ 48h**	**48h**	64.24±1.92	3.56±0.56	32.21±0.66	6.49±1.25
	**72h**	69.63±2.40	3.08±0.40	27.29±0.61	6.52±1.17
**LEN-NDRG2 48h**	**48h**	71.40±0.37	8.50±0.47	20.10±0.30	10.25±2.43
	**72h**	77.61±0.99	7.05±0.15	15.34±0.17	12.88±3.01

Data are presented as % (Mean ± SD, n = 5). Statistical significance was evaluated with ANOVA and SNK tests. *P < 0.01, compared with the controls.

**Figure 8:**
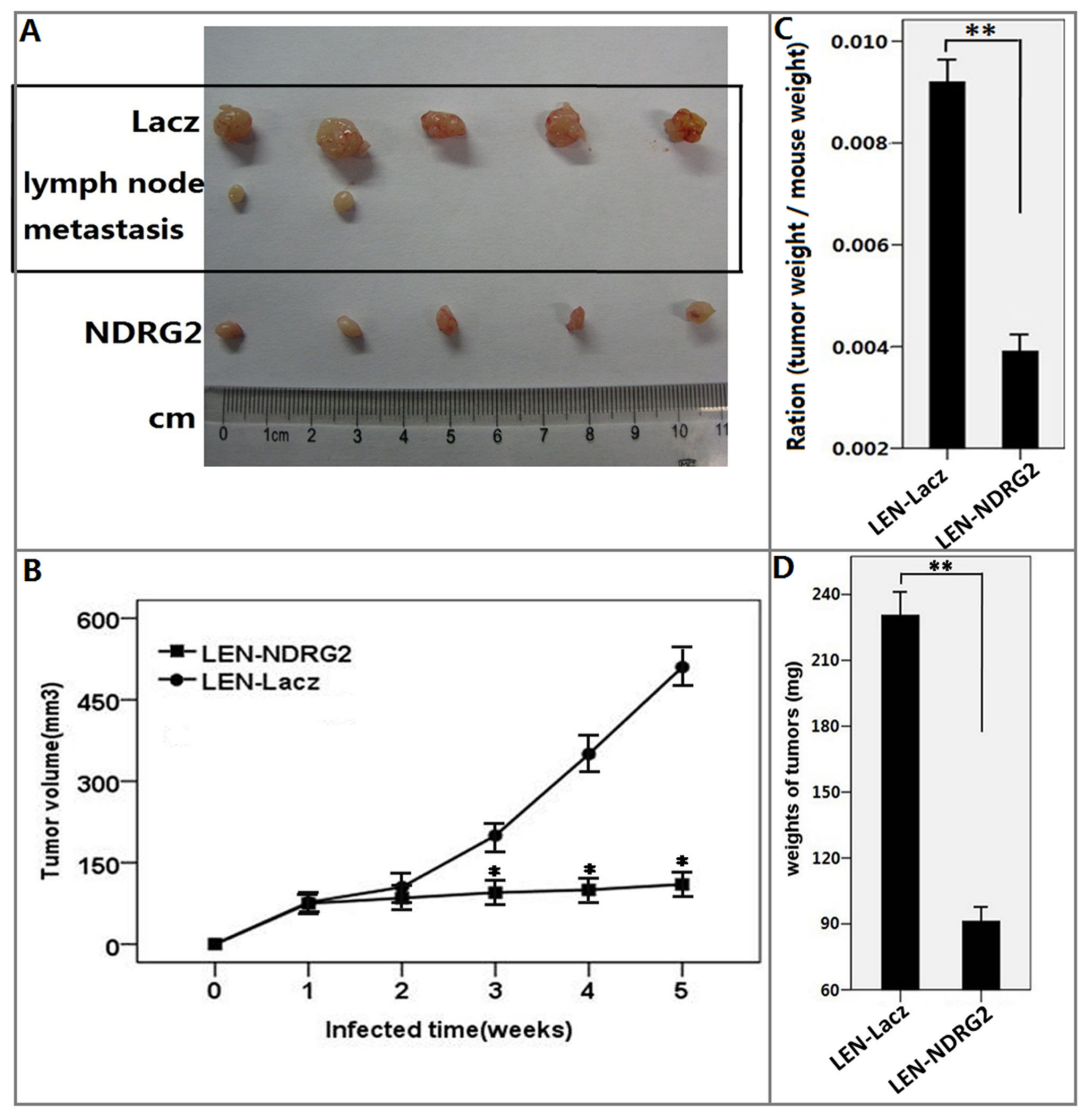
LEN-*NDRG2* can suppress the growth of T24 xenografts *in vivo*. (A) LEN-*NDRG2* suppressed the growth of tumors compared to LEN-LacZ. In addition to unsuppressed tumor growth, the LEN-LacZ group was found to have lymph node metastases. (B) Tumor growth curves. (C) Ratios of tumor masses to mouse masses in different groups. (D) Masses of tumors in two groups. The curves and histograms were drawn based on the average values (n = 5) in two groups. The results are shown as the mean ± SD (*, ** p<0.001).

It was shown by fluorescence microscopy that the efficiency of infection was high being more than 95% (Figure 3A). Real-time PCR analysis showed that the mRNA expression levels of *NDRG2* in the LEN-*NDRG2* group was significantly higher than that in the LEN-Lacz(negative group) and CON groups (*P*<0.05) (Figure 3B). Western blot analysis showed that the *NDRG2* protein level in the LEN-*NDRG2* group was higher than that in the LEN-Lacz and CON groups (Figure 3C). The LEN-*NDRG2* also caused overexpression of *NDRG2* in T24 cells. The cell growth curves and colony formation showed that overexpression of *NDRG2* could suppress the growth of T24 cells more than that of in the other two groups. In the cell growth curves assays, the inhibition began at the third day and became more obviously thereafter (F≥44.58, *P*< 0.01) (Figure 4). The FCA showed that the T24 cells with LEN-*NDRG2* could increase the proportion of cells in G_0_ /G_1_ compared to the LEN-LacZ groups and negative control, indicating that the ovexpression of *NDRG2* had made the T24 cells to arrest in G1 cycle (48 h: F = 41.92, *P* < 0.01; 72 h: F = 24.11, *P* < 0.01). (Figure 5, Table 3.) The western blot showed that overexpression of *NDRG2* downregulated CDK4 and cyclinD1, while upregulate p21 in T24 cells (Figure 6a, b).

**Figure 9:**
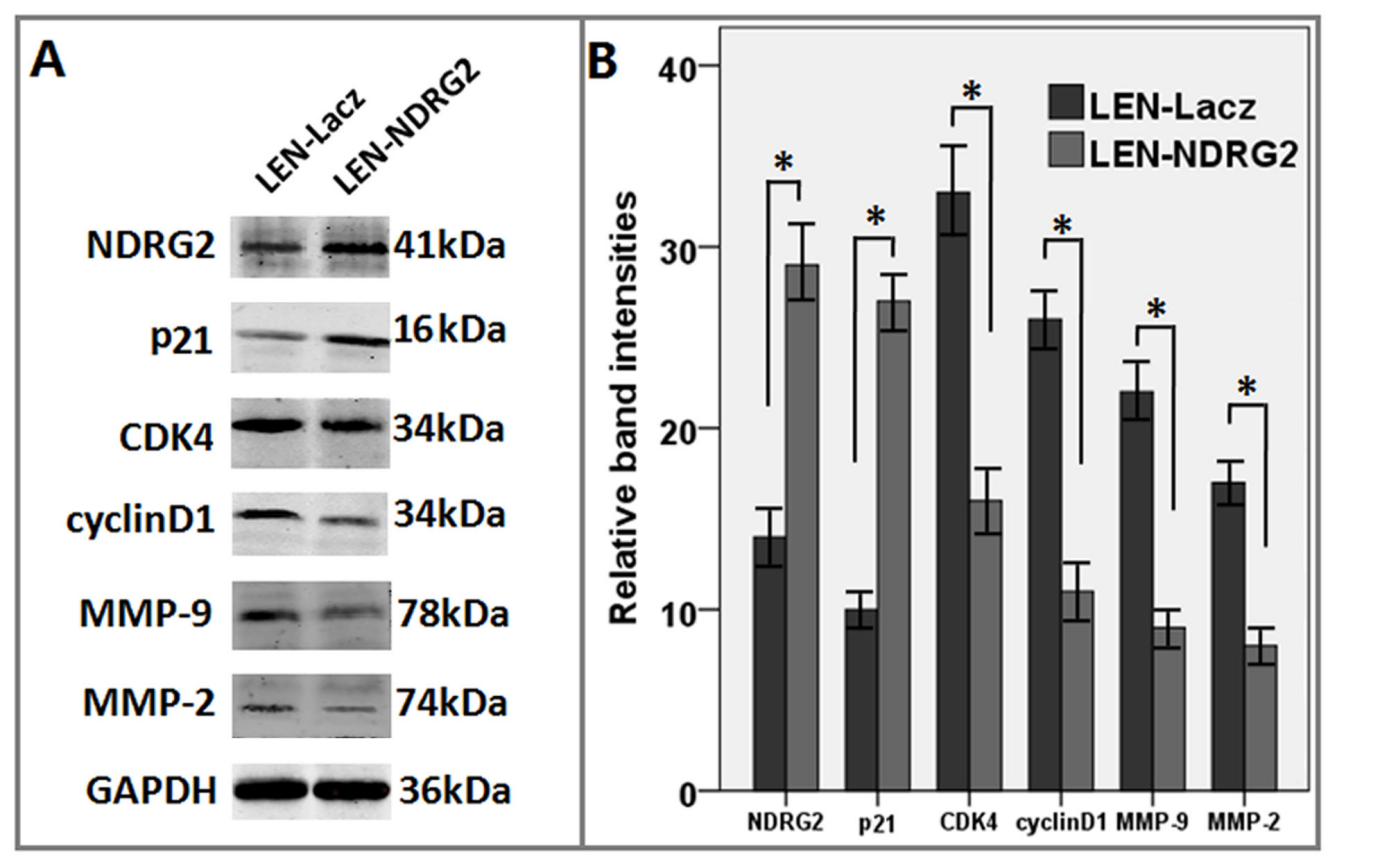
The influences of *NDRG2* overexpression on T24 cells infected with lentivirus *in vivo*. (A) The effect of *NDRG2* overexpression on cell cycle, apoptosis and metastasis regulators as assayed by western blot. (B) Relative quantification of protein expression, normalized to GAPDH levels. Western blots were analyzed with Kodak Digital Science one-dimensional software. The figure shows the tendency of each group as indicated. All the assays were repeated independently for at least three times. The results are shown as the mean ± SD (* p<0.01).

Transwell migration assays and Matrigel-coated transwell invasion assays were performed with T24 cells. The representative micrographs were taken from the lower surface of the transwell filter, and cells which migrated or invaded were stained with Kristallviolet. As shown in Figure 7A, the invasive potential, which is determined by the cells’ ability to invade a Matrigel barrier, was also considerably suppressed in *NDRG2*-overexpressing cells (*P* <0.01). Furthermore, forced expression of *NDRG2* in T24 cells significantly suppressed their migration through the transwell (P < 0.01) (Figure 7B).

In western blot assays, compared with the LEN-Lacz group and blank controls, the group of LEN-*NDRG2* can suppress MMP-2, MMP-9 protein expression level in T24 cell (Figure 7C, D). MMP-2 and MMP-9 were highly expressed in bladder cancer metastasis, so the *NDRG2* likely inhibited the metastasis of bladder cancer by regulating the expression of MMP-2 and MMP-9.

The tumor growth curves was drawn by measuring the size of tumors. All the nude mice survived after injections with lentivirus. The T24 cells were injected 2 weeks later, the tumor’s volume of two groups began to appear differently. From three weeks, the difference in tumor volume started to show statistical significant (F≥31.65, p<0.001) (Figure 8B). Compared with the LEN-Lacz group, the tumor’s weight by LEN-*NDRG2* was significantly lighter (F≥39.82, p<0.001) (Figure 8D). The ratio of tumor weight to mouse weight indicated that the physical condition of the mice in LEN-*NDRG2* group was much better than that of the mice in LEN-LacZ group (F = 39.81, p < 0.001) (Figure 8C), indicating that the overexpression of *NDRG2* could suppress the growth of tumors. Upon anatomizing the mice, ipsilateral lymph node metastases were shown in the LEN-LacZ group, but no other organs were involved; however, in the LEN-*NDRG2* group no any metastatic lesion was found (Figure 8A). Western blot showed that the *NDRG2* protein was upregulated in the tumors of LEN-*NDRG2* group, and that overexpression of *NDRG2* downregulated cyclinD1, CDK4, MMP-2, MMP-9; and upregulated p21 *in vivo*(Figure 9A, B).

## Discussion

Approximately 75% of all bladder cancer patients recur within the first 5 years [14], this high rate of recurrence of bladder cancer and its significant metastatic potential have made patients’ care and prognosis challengeable. Therefore, to increase early diagnosis rate and also to establish novel therapeutic approaches are urgently needed through research of bladder cancer. *NDRG2* is a candidate tumor suppressor gene, and a series of studies have confirmed that it plays an important role in cell proliferation, apoptosis and metastasis [15,16]. Our results in the current study obtained from bladder cancer support these early findings.

We demonstrated that *NDRG2* plays an important role in bladder cancer that is similar to its role in other malignant tumors. Immunohistochemistry results showed that the positive expression level of *NDRG2* in bladder carcinoma tissues was significantly lower than that in normal bladder tissues. The expression levels of *NDRG2* decreased as the degree of bladder carcinoma malignancy increased. We also found that the expression of *NDRG2* was inversely correlated with c-Myc, similar to that seen in other tumor cells. Meanwhile, the *NDRG2* expression levels in bladder cancer cell lines (T24, 5637 and BIU-87) were lower than normal bladder cell line (SV-HUC-1).

Lentiviruses have been shown to be suitable for gene therapy, because they can steady integrate into the host genome to provide long term expression of target gene, in addition, lentiviruses have been confirmed to be progressively safe by the development of split plasmid systems for vector production to prevent generation of replication-competent virus [17].

Investigations carried out by Liu et al. indicated that *NDRG2* was a new target gene that is regulated by p53 and *NDRG2* mRNA and protein levels can be up-regulated in a p53-dependent manner. And they further reported that silencing of *NDRG2* attenuates p53-mediated apoptosis [18]. Meanwhile, it was reported that *NDRG2* could up-regulate the expression of p53 in breast cancer cells [19]. These data strongly suggested that *NDRG2* was an important factor in regulating tumor cell apoptosis. Meanwhile, the CDC20 was regulated by p53 to inhibit the malignant cancer cells grow [20]. Cyclin D1 plays an important role in the cell cycle, binds to cyclin-dependent kinases (CDK4/6), and promotes phosphorylation of RB1, orchestrating progression through the G1 restriction point [21]. *NDRG2* may regulate the expression of proteins (p21, cyclinD1 and CDK4) in bladder cancer cells through up-regulation of p53. We used lentivirus-mediated *NDRG2* overexpression strategy to inhibit the proliferation of T24 cells, to promote their apoptosis both *in vitro* and *in vivo*; this mechanism has been demostracted by regulating the expression of proteins p21, cyclinD1 and CDK4, which are important in cell apoptosis and cycle.

Recently, several reports suggest that *NDRG2* might play a vital role in inhibition of tumor metastasis. It is reported that *NDRG2* could antagonize transforming growth factorβ1-mediated tumor cell invasion by specifically down-regulating the expression of matrix metalloproteinase 2 and laminin 332 pathway components, with concomitant suppression of Rho GTPase activity in hepatocellular carcinomas [22]. The MMP-2 and MMP-9 were high expression in muscle-invasive bladder cancer and metastatic bladder cancer [21,23]. In this study, western blot assay showed that LEN-*NDRG2* in T24 cells was associated with significant reduction in MMP-9 expression and slightly reduced MMP-2 expression suggesting that *NDRG2*-overexpression may regulate the expression of MMP-2 and MMP-9 and inhibit the invasion ability of metastatic bladder cancer cells. This conclusion was supported by both *in vivo* and *in vitro* assays. We suspected that the expression of *NDRG2* significantly suppresses the MMP-2 and MMP-9 by regulating the NF-kB signaling in the bladder cancer cells [24].

In conclusion, we have demonstrated the antioncogenic role of *NDRG2* in the development and invasion of bladder cancer. Since *NDRG2* is implicated in many aspects of tumor progression, including cell growth, cell cycle regulation, invasion and migration, it represents a promising therapeutic target for bladder cancer. However, more investigations are still needed to elucidate further the mechanism of *NDRG2*. Research in this direction will finally provide new methods for early diagnosis, novel therapy and postoperative monitor in bladder cancer.

## References

[1] Jemal A, Bray F, Center MM, Ferlay J, Ward E et al. (2011) Global cancer statistics. CA Cancer J Clin 61: 69-90. doi:10.3322/caac.20107. PubMed: 21296855. 21296855

[2] Jemal A, Siegel R, Ward E, Hao Y, Xu J et al. (2009) Cancer statistics, 2009. CA Cancer J Clin 59: 225-249. doi:10.3322/caac.20006. PubMed: 19474385. 19474385

[3] Boyle H, Fléchon A, Droz JP (2011) Treatment of uncommon malignant tumours of the bladder. Curr Opin Urol 21: 309-314. doi:10.1097/MOU.0b013e3283495758. PubMed: 21814051. 21814051

[4] Zhou RH, Kokame K, Tsukamoto Y, Yutani C, Kato H et al. (2001) Characterization of the human NDRG gene family: a newly identified member, NDRG4, is specifically expressed in brain and heart. Genomics 73: 86-97. doi:10.1006/geno.2000.6496. PubMed: 11352569. 11352569

[5] Yu C, Wu G, Dang N, Zhang W, Zhang R et al. (2011) Inhibition of N-myc downstream-regulated gene 2 in prostatic carcinoma. Cancer Biol Ther 12: 304-313. doi:10.4161/cbt.12.4.16382. PubMed: 21623166. 21623166

[6] Gao L, Wu GJ, Liu XW, Zhang R, Yu L et al. (2011) Suppression of invasion and metastasis of prostate cancer cells by overexpression of NDRG2 gene. Cancer Lett 310: 94-100. doi:10.1016/j.canlet.2011.06.015. PubMed: 21741166. 21741166

[7] Zhao H, Zhang J, Lu J, He X, Chen C et al. (2008) Reduced expression of N-Myc downstream- regulated gene 2 in human thyroid cancer. BMC Cancer 8: 303. doi:10.1186/1471-2407-8-303. PubMed: 18940011. 18940011 PMC2576469

[8] Lorentzen A, Vogel LK, Lewinsky RH, Saebø M, Skjelbred CF et al. (2007) Expression of NDRG2 is down-regulated in high-risk adenomas and colorectal carcinoma. BMC Cancer 7: 192. doi:10.1186/1471-2407-7-192. PubMed: 17935612. 17935612 PMC2099434

[9] Lusis EA, Watson MA, Chicoine MR, Lyman M, Roerig P et al. (2005) Integrative genomic analysis identifies NDRG2 as a candidate tumor suppressor gene frequently inactivated in clinically aggressive meningioma. Cancer Res 65: 7121-7126. doi:10.1158/0008-5472.CAN-05-0043. PubMed: 16103061. 16103061

[10] Hu XL, Liu XP, Lin SX, Deng YC, Liu N et al. (2004) NDRG2 expression and mutation in human liver and pancreatic cancers. World J Gastroenterol 10: 3518-3521. PubMed: 15526377. 15526377 10.3748/wjg.v10.i23.3518 PMC4576239

[11] Deng Y, Yao L, Chau L, Ng SS, Peng Y et al. (2003) N-Myc downstream-regulated gene 2 (NDRG2) inhibits glioblastoma cell proliferation. Int J Cancer 106: 342-347. doi:10.1002/ijc.11228. PubMed: 12845671. 12845671

[12] Foletta VC, Prior MJ, Stupka N, Carey K, Segal DH et al. (2009) NDRG2, a novel regulator of myoblast proliferation, is regulated by anabolic and catabolic factors. J Physiol 587: 1619-1634. doi:10.1113/jphysiol.2008.167882. PubMed: 19204049. 19204049 PMC2678230

[13] Phillips HS, Kharbanda S, Chen R, Forrest WF, Soriano RH et al. (2006) Molecular subclasses of high-grade glioma predict prognosis, delineate a pattern of disease progression, and resemble stages in neurogenesis. Cancer Cell 9: 157-173. doi:10.1016/j.ccr.2006.02.019. PubMed: 16530701. 16530701

[14] Mansoor M, Ali S, Fasihuddin Q, Baloch MU (2011) Superficial bladder tumours: recurrence and progression. J Coll Physicians Surg Pak 21: 157-160. PubMed: 21419022. 21419022

[15] Yao L, Zhang J, Liu X (2008) NDRG2: a Myc-repressed gene involved in cancer and cell stress. Acta Biochim Biophys Sin (Shanghai) 40: 625-635. doi:10.1111/j.1745-7270.2008.00434.x. PubMed: 18604454. 18604454

[16] Kim A, Kim MJ, Yang Y, Kim JW, Yeom YI et al. (2009) Suppression of NF-kappaB activity by NDRG2 expression attenuates the invasive potential of highly malignant tumor cells. Carcinogenesis 30: 927-936. doi:10.1093/carcin/bgp072. PubMed: 19336468. 19336468

[17] Manjunath N, Wu H, Subramanya S, Shankar P (2009) Lentiviral delivery of short hairpin RNAs. Adv Drug Deliv Rev 61: 732-745. doi:10.1016/j.addr.2009.03.004. PubMed: 19341774. 19341774 PMC2789654

[18] Liu N, Wang L, Li X, Yang Q, Liu X et al. (2008) N-Myc downstream-regulated gene 2 is involved in p53-mediated apoptosis. Nucleic Acids Res 36: 5335-5349. doi:10.1093/nar/gkn504. PubMed: 18689861. 18689861 PMC2532733

[19] Ma J, Liu W, Yan X, Wang Q, Zhao Q et al. (2012) Inhibition of endothelial cell proliferation and tumor angiogenesis by up-regulating NDRG2 expression in breast cancer cells. PLOS ONE. 7: e32368. doi:10.1371/journal.pone.0032368. PubMed: 22393400. 22393400 PMC3290656

[20] Kidokoro T, Tanikawa C, Furukawa Y, Katagiri T, Nakamura Y et al. (2008) CDC20, a potential cancer therapeutic target, is negatively regulated by p53. Oncogene 27: 1562-1571. doi:10.1038/sj.onc.1210799. PubMed: 17873905. 17873905

[21] Zaravinos A, Lambrou GI, Boulalas I, Delakas D, Spandidos DA (2011) Identification of common differentially expressed genes in urinary bladder cancer. PLOS ONE 6: e18135. doi:10.1371/journal.pone.0018135. PubMed: 21483740. 21483740 PMC3070717

[22] Lee DC, Kang YK, Kim WH, Jang YJ, Kim DJ et al. (2008) Functional and clinical evidence for NDRG2 as a candidate suppressor of liver cancer metastasis. Cancer Res 68: 4210-4220. doi:10.1158/0008-5472.CAN-07-5040. PubMed: 18519680. 18519680

[23] Szarvas T, Vom DF, Ergun S, Rubben H (2011) Matrix metalloproteinases and their clinical relevance in urinary bladder cancer. Nat. Rev Urol 8: 241-254. doi:10.1038/nrurol.2011.44. 21487384

[24] Kim A, Kim MJ, Yang Y, Kim JW, Yeom YI et al. (2009) Suppression of NF-kappaB activity by NDRG2 expression attenuates the invasive potential of highly malignant tumor cells. Carcinogenesis 30: 927-936. doi:10.1093/carcin/bgp072. PubMed: 19336468. 19336468

